# Continuous symmetry measures: From point groups to space groups

**DOI:** 10.1063/4.0001024

**Published:** 2025-10-27

**Authors:** Gil Alon Senior Lecturer

**Affiliations:** The Open University of Israel, Raanana, Israel, Israel

## Abstract

The CSM (Continuous Symmetry Measure) was developed in the 1990s by Avnir, Peleg and Zabrodsky as a measure for the symmetry level of molecules ([1]).

For a given molecular structure, the calculation of the CSM is a nontrivial task. Over the past 8 years, we have developed exact and approximate algorithms for the calculation of the CSM in various conditions, depending on the size and type of the molecules ([2]-[5]).

It is natural to try to extend these methods to the symmetry analysis of crystals, where we could have a continuous scale on which the fit of a given crystal to a given space symmetry group will be measured.

In this talk I will describe the algorithms we developed for molecules, and our work in progress towards extending these methods to the symmetry analysis of crystals.

Joint work with Inbal Tuvi-Arad.
